# Effect of lidocaine on intraoperative blood pressure variability in patients undergoing major vascular surgery

**DOI:** 10.1186/s12871-024-02550-5

**Published:** 2024-05-07

**Authors:** Dariusz Gajniak, Konrad Mendrala, Gabriela König-Widuch, Szymon Parzonka, Danuta Gierek, Łukasz J Krzych

**Affiliations:** 1 Department of Anaesthesiology and Intensive Care, Upper-Silesian Medical Centre of the Medical University of Silesia in Katowice, Ziolowa 45 street, Katowice, 40-635 Poland; 2 Department of Anaesthesiology and Intensive Care, Faculty of Medical Sciences in Katowice, Medical University of Silesia, Katowice, Poland; 3 Department of Acute Medicine, Faculty of Medical Sciences in Zabrze, Medical University of Silesia, Zabrze, Poland

**Keywords:** Blood pressure variability, Aorta, High-risk surgery, Lidocaine, Vascular surgery, Intraoperative

## Abstract

**Background:**

Dynamic fluctuations of arterial blood pressure known as blood pressure variability (BPV) may have short and long-term undesirable consequences. During surgical procedures blood pressure is usually measured in equal intervals allowing to assess its intraoperative variability, which significance for peri and post-operative period is still under debate. Lidocaine has positive cardiovascular effects, which may go beyond its antiarrhythmic activity. The aim of the study was to verify whether the use of intravenous lidocaine may affect intraoperative BPV in patients undergoing major vascular procedures.

**Methods:**

We performed a post-hoc analysis of the data collected during the previous randomized clinical trial by Gajniak et al. In the original study patients undergoing elective abdominal aorta and/or iliac arteries open surgery were randomized into two groups to receive intravenous infusion of 1% lidocaine or placebo at the same infusion rate based on ideal body weight, in concomitance with general anesthesia. We analyzed systolic (SBP), diastolic (DBP) and mean arterial blood (MAP) pressure recorded in 5-minute intervals (from the first measurement before induction of general anaesthesia until the last after emergence from anaesthesia). Blood pressure variability was then calculated for SBP and MAP, and expressed as: standard deviation (SD), coefficient of variation (CV), average real variability (ARV) and coefficient of hemodynamic stability (C10%), and compared between both groups.

**Results:**

All calculated indexes were comparable between groups. In the lidocaine and placebo groups systolic blood pressure SD, CV, AVR and C10% were 20.17 vs. 19.28, 16.40 vs. 15.64, 14.74 vs. 14.08 and 0.45 vs. 0.45 respectively. No differences were observed regarding type of surgery, operating and anaesthetic time, administration of vasoactive agents and intravenous fluids, including blood products.

**Conclusion:**

In high-risk vascular surgery performed under general anesthesia, lidocaine infusion had no effect on arterial blood pressure variability.

**Trial registration:**

ClinicalTrials.gov; NCT04691726 post-hoc analysis; date of registration 31/12/2020.

## Introduction

Arterial blood pressure (BP) is a highly variable parameter [1]. It has many patient-related and environmental determinants, therefore not only single measurements, but also assessment of its fluctuation know as blood pressure variability (BPV) is of great interest in everyday clinical practice [2–4]. Both day-to-day (mid-term) or visit-to-visit (long-term) BPV are associated with long-term negative consequences, such as increased risk of cardiovascular events, subclinical organ damage, mortality, microalbuminuria and progression of renal failure [4]. The occurrence of BP fluctuations and its significance in the very-short-term BPV (beat-to-beat) is definitely more difficult to interpret, because of the interaction between numerous external and internal factors such as neuronal, humoral, vascular, cardiac, rheological, renal, metabolic, environmental, emotional and behavioural [5].

Intraoperative BP variability is still a subject of research, the interpretation of which encounters problems related to the lack of clear definitions and using different definitions by different authors [5, 6]. Compared to the results of variability obtained in the general population, patients undergoing general anaesthesia are subjected to higher blood pressure variability [7]. Tissue trauma associated with the surgical procedure causes the nociceptive stimuli and activate sympathetic nervous system increasing heart rate (HR) and blood pressure [8]. .

Most common management in response to nociceptive stimulus is based on multimodal analgesia concept, combining various groups of medications for pain relief. As opioids remain the main treatment for severe postoperative pain but not free of severe side effects, adding different drugs increase the effectiveness of analgesic strategy and may allow opioid sparing analgesia (OSA) or even opioid free analgesia (OFA) [9].

One of the drugs used in multimodal analgesia is lidocaine, administered as a continuous intravenous infusion. Its effectiveness was demonstrated mainly in abdominal surgery including major vascular surgery [10, 11]. Therefore, we sought to verify whether the use of intravenous lidocaine may affect intraoperative BPV in patients undergoing major vascular procedures performed using the classical (open) technique. We assessed SBP variability as most studies show a close relationship with outcomes, especially in the elderly [12, 13]. In addition, MAP was analysed due to its potential clinical relevance, particularly in patients monitored by the automatic, non-invasive blood pressure monitor DINAMAP (Device for Indirect Non-invasive Mean Arterial Pressure).

This is a secondary analysis of our primary study on efficacy and safety of lidocaine in this clinical scenario [11].

## Methods

We performed a post-hoc analysis of the data collected during the previous randomized clinical trial by Gajniak et al. carried out at the university hospital in Poland between February 2019 and July 2022 [11]. The original study was approved by the Medical University of Silesia’s Ethics Committee in Katowice, Poland (KNW/0022/KB1/1/19) and registered at ClinicalTrials.gov (registration number NCT04691726).

In the original study patients undergoing elective abdominal aorta and/or iliac arteries open surgery were randomized into two groups to receive intravenous infusion of 1% lidocaine or placebo at the same infusion rate based on ideal body weight, in concomitance with general anesthesia (GA). Induction of anesthesia was performed with propofol, atracurium and fentanyl administered intravenously. Anesthesia was maintained with desflurane and repeated doses of muscle relaxants.

As per original study protocol systolic, diastolic and mean arterial blood pressure was measured invasively through catheter in radial artery and recorded in 5-minutes intervals (from the first measurement before induction of general anaesthesia until the last after emergence from anaesthesia) using Datex-Ohmeda F-CM1-05 (GE Healthcare, Helsinki, Finland). All data was recorded by a computer connected to the vital signs monitor in a real-time manner using dedicated software (Vital Signs Capture Wave v1.006; 2018 John George K.).

Based on the data collected from the original study for each patient’s blood pressure records, we calculated standard deviation (SD), coefficient of variation (CV), average real variability (ARV), and coefficient of hemodynamic stability (C10%) [6, 14].

Coefficient of hemodynamic stability (C10%) was an index designed by our research team. Consecutive 5-minutes blood pressure values were compared value to the next value. Each time the difference was exceeding 10% of the previous value 1 point was scored. In the next step coefficient was calculated as: total number of exceedances divided by total number of measurements taken for each patient. The primary outcome of the post hoc-analysis was the intraoperative blood pressure variability comparison expressed in the form of the indices presented above. Additionally, we conducted a comparison of variability in selected diseases such as hypertension, peripheral artery disease and smoking habit.

### Statistical analysis

For statistical analysis, we used Jamovi 2.3.28 software [15]. The distribution of variables was assessed with the Shapiro–Wilk test and QQ plots. Quantitative variables were presented as the mean and standard deviation (SD) or median and interquartile range (IQR, interquartile range). Qualitative variables were presented as absolute values and percentages. Differences between groups were assessed using the Student’s t test or Mann–Whitney U test. For qualitative variables, contingency tables and the chi-square test were used. We assumed *p* < 0.05 to be statistically significant.

.

**Table 1:** Characteristics of patients

	Lidocaine (*n* = 32)	Placebo (*n* = 35)	*p*-Value
Age; [y] median (IQR)	64.4 (62.4–66.4)	64.5 (62.4–67)	0.91
Male; n (%)	28 (88%)	28 (80%)	0.4
BMI; mean (95% CI)	26.4 (24.9–27.9)	27.2 (25.7–28.7)	0.46
Smoking habit; n (%)	20 (62%)	20 (57%)	0.8
Hypertension; n (%)	29 (90%)	28 (80%)	0.22
Preoperative medications:			
ACE-I/ARBs; n (%)	19 (59%)	19 (54%)	0.67
β-blockers; n (%)	15 (47%)	19 (54%)	0.54
CCBs; n (%)	6 (19%)	13 (37%)	0.09
Diuretics; n (%)	7 (22%)	5 (14%)	0.42

IQR - Inter-quartile range, 95% CI – 95% confidence interval, BMI – body mass index, ACEI – angiotensin-converting enzyme inhibitors; ARBs – angiotensin receptor blockers; CCBs- calcium channel blockers

**Table 2:** Comparison of the intraoperative vasoactive agent’s administration and fluid balance

	Lidocaine (*n* = 32)	Placebo (*n* = 35)	*p*-Value
**Use of intraoperative vasoactive agents**			
Norepinephrine; n (%)	9 (28%)	9(26%)	0.82
Ephedrine; n (%)	27 (84%)	31(88%)	0.61
Ephedrine dose; median (IQR)	25 mg (10–25)	20 mg (12,5–25)	0.58
Atropine; n (%)	18 (56%)	17 (48%)	0.53
Atropine dose (min.-max.)*	0,5 − 1,0 mg	0,5 − 1,5 mg	0.34
Urapidil; n (%)	2 (6%)	5 (14%)	0.28
Urapidil dose; (min.-max.)*	5-10 mg	20-25 mg	-
**Intraoperative fluid balance**			
Total intravenous fluids; mean (95% CI)	2845 ml (2675–3015)	3073 ml (2841–3305)	0.12
Colloids; n (%)	19 (59%)	18 (51%)	0.51
Estimated blood loss; median (IQR)	550 ml (400–725)	700 ml (500–900)	0.19
Blood transfusions; n (%)	0 (0%)	3 (9%)	0.09

* due to the small number of interventions, minimum and maximum doses are given

**Table 3:** Comparison of blood pressure variation indexes. Continuous data were presented as median (IQR) or mean value (95% CI); ^#^ t-student test

	Lidocaine (*n* = 32)	Placebo (*n* = 35)	*p*-Value
SD SBP	20.17 (18.62–21.71)	19.275 (17.57–20.98)	0.43
CV SBP	16.40 (15.29–17.51)	15.64 (14.30-16.98)	0.38
ARV SBP	14.74 (11.5-17.86)	14.08 (12.26–17.73)	0.83
C10% SBP	0.45 (0.41–0.48)	0.45 (0.42–0.49)	0.78^#^
SD MAP	13.52 (12.74–14.30)	12.83 (11.71–13.94)	0.31
CV MAP	15.57 (14.75–16.38)	14.73 (13.43–16.03)	0.28
ARV MAP	9.65 (8.60-11.47)	9.81 (8.26–11.28)	0.80
C10% MAP	0.44 (0.40–0.47)	0.44 (0.40–0.48)	0.92^#^

**Table 4:** Sub-analysis of indices in different subgroups. Categorical data are presented as total number and %. Indices are presented as mean value (95%CI) or median (IQR). ^#^ U Mann-Whitney test

	Lidocaine (*n* = 32)	Placebo (*n* = 35)	*p* – Value
**Hypertension n (%)**	29 (90%)	28 (80%)	
**ARV SBP**	15.0 (13.4–16.5)	15.6 (13.8–17.4)	0.589
**SD SBP**	20.2 (18.6–21.9)	19.8 (17.8–21.9)	0.73
**CV SBP**	16.4 (15.3–17.6)	16.1 (14.5–17.7)	0.717
**ARV MAP**	9.4 (8.7–11.7)	9.7 (8.2–11.4)	0.798^#^
**SD MAP**	13.7 (12.8–14.5)	13.1 (11.7–14.4)	0.445
**CV MAP**	15.6 (14.8–16.5)	15.1 (13.6–16.7)	0.559
**Smoking habit n (%)**	20 (62%)	20 (57%)	
**ARV SBP**	15.0 (13.2–16.8)	15.8 (13.5–18.2)	0.562
**SD SBP**	19.5 (17.7–21.3)	19.2 (16.6–21.7)	0.827
**CV SBP**	16.2 (14.8–17.5)	15.4 (13.4–17.3)	0.471
**ARV MAP**	10.2 (9.2–11.2)	9.8 (8.7–11.4)	0.825^#^
**SD MAP**	13.3 (12.4–14.2)	12.8 (11.1–14.5)	0.547
**CV MAP**	15.8 (13.3–17.7)	14.5 (10.9–18.3)	0.368^#^
**PAD n (%)**	16 (50%)	17 ( 48%)	
**ARV SBP**	15.9 (14.0-17.7)	15.4 (13.4–17.4)	0.717
**SD SBP**	21.1 (17.7–23.7)	19.8 (17.6–22.1)	0.427
**CV SBP**	16.8 (14.7–18.8)	16.4 (14.7–18.0)	0.747
**Preoperative medications**:			
**ACE-I/ARBs**	19 (59%)	19 (54%)	
**ARV SPB**	14.4 (12.7–16.1)	15.4 (13.4–17.4)	0.43
**SD SPB**	20.1 (17.9–22.3)	20.1 (17.6–22.6)	0.988
**CV SBP**	16.6 (15.1–18.2)	16.6 (14.6–18.6)	0.969
**β-blockers**	15 (47%)	19 (54%)	
**ARV SBP**	15.3 (12.9–17.7)	14.0 (12.3–15.7)	0.33
**SD SBP**	21.3 (18.6–24.0)	18.1 (15.7–20.4)	0.064
**CV SBP**	15.9 (15.6–18.7)	15.6 (12.6–16.7)	0.061^#^
**CCBs**	6 (19%)	13 (37%)	
**ARV SBP**	16.2 ( 12.3–20.0)	13.1(11.3–14.9)	0.066
**SD SBP**	20.3 (17.0-23.7)	17.6 (14.6–20.6)	0.227
**CV SBP**	17.0 (15.3–18.7)	14.9 (12.2–17.5)	0.261
**HA + ACE-I/ARBs**	19 (59%)	19 (54%)	
**ARV SBP**	14.4 (12.7–16.1)	15.4 (13.4–17.4)	0.431
**SD SBP**	20.1 (17.9–22.3)	20.1 (17.6–22.6)	0.988
**CV SBP**	16.6 (15.1–18.2)	16.6 (14.6–18.6)	0.969
**HA + β-blockers**	15 (47%)	16 (46%)	
**ARV SBP**	15.3 (12.9–17.7)	14.0 (12.1–15.9)	0.366
**SD SBP**	21.3 (18.6–24.0)	18.3 (15.4–21.1)	0.106
**CV SBP**	15.9 (15.6–18.7)	15.6 (11.4–17.4)	0.128^#^

PAD – peripheral artery disease, ACE-I – angiotensin-converting enzyme inhibitor; ARBs – angiotensin receptor blockers; CCBs- calcium channel blockers; HA - hypertension

## Results

The data collected from the study by Gajniak et al. involved patients undergoing subrenal major aortic surgery [11]. In the original study no differences were observed between the groups considering comorbidities, surgical techniques and depth of anesthesia. The detailed information on patient characteristics and antihypertensive medication are shown in Table 1. In our post hoc analysis no differences were observed between the groups regarding intraoperative use of vasoactive agents, fluid therapy and blood loss. Details are shown in Table 2. For lidocaine and placebo groups, the fluctuation indices SD, CV, ARV and CD10% for systolic blood pressure were 20.2 vs. 19.3, 16.4 vs. 15.6, 14.7 vs. 14.1, 0.5 vs. 0.5, respectively, and did not differ statistically. Also, for mean arterial pressure, no differences were found (Table 3).

The sub-analysis of variability performed for selected criteria between the lidocaine and placebo groups also did not show statistically significant differences (Table 4).

## Discussion

Our study is the first publication on intraoperative pressure variability among patients undergoing high-risk cardiac vascular surgery (i.e. risk exceeding 5%, according to the ESC classification), which allowed us to compare the obtained indices with data from the literature on values obtained in other types of surgical procedures. We found no effect of lidocaine infusion on intraoperative systolic blood pressure variability.

To date, there is no clear consensus on which indices should be used to determine intraoperative variability of blood pressure, which can significantly affect both the possibility of making comparisons between tests and the interpretation of results [16]. For this reason, we decided to calculate most popular indices. Unambiguous interpretation and comparison of data is also hindered by the fact that each of the indexes can be calculated for SBP, DBP and MAP values. Also, numerous papers indicate the reasons for obtaining a different result in identical sets of measurements when calculating different indexes variants [12, 16, 17]. Moreover, as shown by Mena et al., a different degree of correlation may be seen between different BPV indexes and the same endpoint [6]. 

Most data on the clinical significance of blood pressure variability come from measurements obtained outside the operating room [18, 19]. A recent meta-analysis by Putowski et al. showed that this type of observation cannot be easily related to the intraoperative period [16]. The work of Levin et al. on a group of almost 53 thousand patients presents different conclusions: increased lability was associated with decreased mortality, which the authors explain as an adaptive response that demonstrates physiological reserve [20]. In our study, we used the C10% index, a modified lability index first described in the cited work [20]. We calculated C10% index by dividing the total number of deviations by the total number of measurements to exclude the influence of the surgical procedure duration.

The study of Wiórek and Krzych analysed the effect of intraoperative BPV expressed in the coefficient of variation index for SBP on 30-day mortality in the postoperative period. Compared to the results of the cited work, the CV index for SBP results obtained by us in the lidocaine group amounted to 16.40 (15.29–17.51), and in the placebo group, 15.64 (14.30-16.98), allowing us to conclude that in vascular surgery the variability is much higher compared to the results presented in the cited work, where the median for gastrointestinal, gynaecological and neurosurgical procedures were 12.32 (9.6-14.64), 9.86 (6.95–13.29) and 12.32 (9.27–16.02) respectively [14]. A comparison of the CV SBP from the cited paper calculated for high-risk cardiac procedures was 12.78 (10.93–15.46) with that obtained in our groups, indicating significantly higher variability in our population. Compared to other surgical specialties, the high variability observed in our study may result from specific surgical techniques such as aortic clamping/declamping and reperfusion syndrome. In addition, we performed the analysis in a subpopulation of patients with potential autonomic dysfunction and possibly increased BPV (with hypertension, peripheral vascular disease, and active smoking) [4]. This comparison also did not reveal any effect of lidocaine infusion on blood pressure variability. Taking into account the relationship between BPV in patients treated for hypertension and the type of antihypertensive drugs used, previous studies have shown that taking amlodipine reduces BPV compared to atenolol. A reduction in variability was also achieved in other studies with amlodipine and diuretics [1]. In the study by Levin et al., based on intraoperative pressure measurements, the most significant variability was observed in the groups taking ACEI/ARBs only, followed by patients taking β-blockers [20]. In our study, the analysis of variability based on the type of antihypertensive medications taken did not show statistically significant differences between the groups. However, it does not allow for a straightforward interpretation, as in both groups, about half of the patients took at least two different antihypertensive drugs, and only a small part was treated with a single medication (eleven patients (34%) in lidocaine and five patients (14%) in placebo groups).

Finally, there are no publications on the effect of multimodal analgesic strategies on blood pressure variability. The mechanism of action of lidocaine administered as an intravenous infusion is not clearly defined, which makes it impossible to draw accurate conclusions. Kawamata et al. suggest that using lidocaine before the surgical stimulus reduces the excessive inputs from the injured peripheral nerves and reduces secondary hyperalgesia [21]. The exact mechanism of action has yet to be elucidated. Still, possible mechanisms are blocking NMDA receptors, acting directly through the opioid receptor, or decreasing excitability and conduction of unmyelinated C fibres [22–25]. However, the literature lacks information on whether multimodal techniques reduce intraoperative BPV and whether the changes in BPV affect long-term results. In some populations, effective analgesic management may lead to a decrease in the activation of the sympathetic nervous system and the variability observed in the circulatory system. However, the true meaning of this phenomenon is still under debate [16, 20]. Confirmation of the validity of such a claim should be sought by assessing hard endpoints performed after consensus regarding methodology.

### Study limitations

The major limitation of our study is the small group of participants and a single-center design, which may not allow us to draw definite conclusions. The method of lidocaine regimen used in our study was based on Polish national guidelines and may differ from other centers. Our study raises the question of short-term BPV in the context of vascular surgery, recognizing that the full spectrum of implications may not be fully understood. The complexity of physiological responses during surgery and the specific impact of short-term BPV on patient outcomes remain areas for further exploration. Also, the 5-minute intraoperative blood pressure recording interval is considered the classic approach to systematically record pressure changes during surgical procedures. Still, it may not capture rapid fluctuations or transient events in shorter time frames. On the other hand, this approach reduces sensitivity to short iatrogenic pressure fluctuations, particularly in vascular surgery. Also, while the study focuses on various aspects of blood pressure variability, excluding DBP may limit the comprehensiveness of the findings. The C10% index was developed for our study and has not been validated.

## Conclusion

In the study population, the intraoperative use of lidocaine infusion as an adjuvant in analgesic treatment did not affect the intraoperative variability of blood pressure expressed by the most commonly used indicators.

## Data Availability

The data generated and analyzed during this study are not publicly available due to the protection for the patients’ privacy, but are available from the corresponding author on the reasonable request.

## Abbreviations

ACE-I: Angiotensin-converting enzyme inhibitor
ARBs: Angiotensin receptor blockers
ASA: American Society of Anesthesiologists
ARV: Average real variability
BPV: Blood pressure variability
BP: Blood pressure
CAD: Coronary artery disease
CCBs: Calcium channel blockers
CKD: Chronic kidney disease
COPD: Chronic obstructive pulmonary disease
CV: Coefficient of variation
C10%: Coefficient of hemodynamic stability
DBP: Diastolic blood pressure
DINAMAP: Device for Indirect Non-invasive Mean Arterial Pressure
ESC: European Society of Cardiology
HA: Hypertension
IBW: Ideal Body Weight
IC: Stroke or transient ischemic attack
IQR: Interquartile range
MAP: Mean arterial blood pressure
MI: Myocardial infraction
OFA: Opioid free analgesia
OSA: Opioid sparing analgesia
PAD: Peripheral artery disease
RE: Response entropy
SBP: Systolic blood pressure
SD: Standard deviation
SE: State entropy
T2DM: Type 2 diabetes mellitus
VQI CRI: Vascular Quality Initiative Cardiac Risk Index
VQI RI: Vascular Quality Initiative Respiratory Adverse Event Post Vascular Surgery
